# GridFF: Efficient Simulation of Organic Molecules on Rigid Substrates

**DOI:** 10.1021/acs.jctc.5c01223

**Published:** 2025-11-26

**Authors:** Indranil Mal, Milan Kočí, Paolo Nicolini, Prokop Hapala

**Affiliations:** † 86889 FZU - Institute of Physics of the Czech Academy of Sciences Na Slovance 2, Prague 18 200, Czech Republic; ‡ Faculty of Nuclear Sciences and Physical Engineering, Czech Technical University in Prague, Břehová 7, Prague 115 19, Czech Republic

## Abstract

We present GridFF, an efficient method for simulating molecules on rigid substrates, derived from techniques used in protein–ligand docking in biochemistry. By projecting molecule–substrate interactions onto precomputed spatial grids with tricubic B-spline interpolation, GridFF reduces the computational cost by orders of magnitude compared to traditional pairwise atomistic models, without compromising the accuracy of forces or trajectories. The CPU implementation of GridFF in the open-source FireCore package provides a 100–1000× speedup over all-atom simulations using LAMMPS, while the GPU implementation – running thousands of system replicas in parallel – samples millions of configurations per second, enabling an exhaustive exploration of the configuration space of small flexible molecules on surfaces within minutes. Furthermore, as demonstrated in our previous application of a similar technique to high-resolution scanning probe microscopy, GridFF can be extended beyond empirical pairwise potentials to those derived from *ab initio* electron densities. Altogether, this unlocks accurate high-throughput modeling of molecular self-assembly, adsorption, and scanning probe manipulation in surface science.

## Graphical abstract

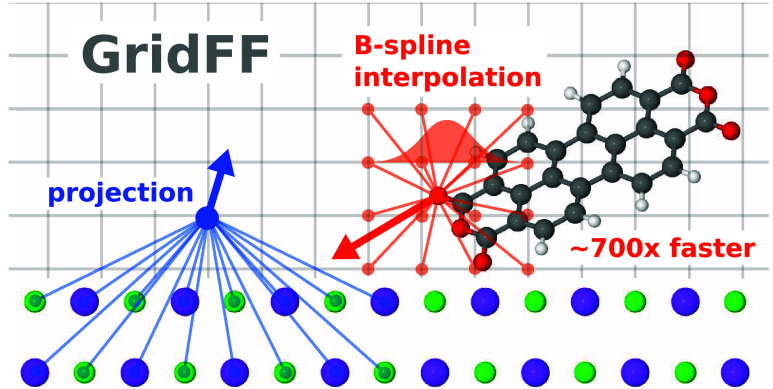

## 1 Introduction

The structural characterization and design of organic/inorganic interfaces represent a critical challenge across multiple research and technological fields, including friction and lubrication,^1–3^ molecular electronics,^4^ photovoltaics,^5^ and scanning probe microscopy (SPM).^6^ The interaction of organic molecules with surfaces of inorganic crystals plays a key role in the development of molecular nanotechnology comprising self-assembled monolayers,^7^ 2D molecular crystals,^8^ covalent organic frameworks,^9^ and also for the emerging field of on-surface chemistry.^10^

Complex molecular nanostructures are one of the most promising building blocks for next-generation computational devices such as molecular electronics,^11^ photonics,^12^ and quantum cellular automata.^13^ The difficulty of designing such structures, especially from flexible molecules, arises from the curse of dimensionality, related to the soft internal degrees of freedom (*e.g.*, torsions along single bonds), which drastically expands their configuration space.

The formation of such structures is primarily governed by noncovalent interactions between organic molecules and the templating effects of the inorganic substrate. Crystalline inorganic substrates provide well-defined supports for constructing and studying complex nanosystems,^14^ effectively restraining the vast configuration space of molecular assemblies. Unlike bulk solids or liquid solutions, surfaces allow unobstructed access to functional molecular components and atomic-level control through SPM techniques, enabling both high-resolution imaging^15^ and direct manipulation of supramolecular structures.^16^

Despite these advantages, predicting the adsorption configurations of molecules on surface and self-assembled layers, as well as achieving precise control of molecular degrees of freedom remains challenging and heavily relies on atomistic simulations; without them, atomic force microscopy (AFM) and/or scanning tunneling microscopy (STM) manipulation is mostly trial-and-error.^17^ Recent efforts to automate this laborious process involve reinforcement learning for robotic AFM/STM machines,^18–20^ but experimental training is prohibitively costly. This underscores the need for a specialized virtual training nanophysics engine – akin to NVIDIA’s Isaac physics engine^21^ – capable of generating the data needed for training robotic manipulation of nanoscale objects, possibly parallelized on graphics processing units (GPU).

Moreover, the structure of molecular assemblies at finite temperatures is influenced not only by enthalpy, but also by entropic contributions. In fact, for flexible molecules, entropy can play a particularly significant role. The temperature dependence of the entropy term in the Gibbs free energy (Δ*G* = Δ*H* – *T*Δ*S*) is crucial for estimating the melting temperature of self-assembled structures and determining annealing conditions for their reversible formation. These are key parameters in computational design, particularly for larger self-assembling molecular templates. However, the computational estimation of entropy and free energy requires an exhaustive configuration sampling of the partition function, which is extremely demanding – especially when one aims at screening a large number of candidate molecules.

Another challenge of simulating organic molecules on inorganic substrates consists of the usage of large supercells comprising a large number of atoms, where the number of substrate atoms *n*_*S*_ (replicated across multiple unit cells) far exceeds the number of atoms of the organic molecules *n*_*M*_ being studied, leading to substantial computational overhead, as usually *n*_*S*_ ≫ *n*_*M*_.^22^ On the other hand, it is a common practice to fix the position of the substrate atoms, or at least the bottom atomic layers. But, even in this case, the computational overhead is not reduced in most implementations as the number of pairwise interactions scales quadratically with the total number of atoms in the supercell (*n*_*M*_ + *n*_*S*_)^2^. Even if pairwise interactions between the atoms of the substrate can be eliminated (which is generally a nonstandard feature in classical force field programs^23^), the computational cost of evaluating *n*_*M*_ *n*_*S*_ pairwise interactions between the molecule and the substrate dominates over the *n*_*M*_^2^ calculations required to describe the interactions between atoms in the molecule.

Consequently, both SPM manipulation of molecules on solid substrates and their self-assembly ultimately face the same fundamental challenge: exploring a vast configuration space while simultaneously describing their interactions with the substrate (and potentially with an AFM/STM tip) efficiently. Unfortunately, many existing methods for molecular configuration exploration, such as CREST^24^ – designed for systems in gas or liquid phases – cannot be directly applied to molecules on surfaces. Likewise, state-of-the-art force fields, like AMBER,^25^ CHARMM,^26^ GROMOS^27^ and OPLS^28^ (which can be used on general-purpose simulation programs such as AMBER,^29^ CHARMM,^30^ GROMACS,^31^ LAMMPS^32^ and NAMD^33^), are primarily optimized for biological molecules in aqueous environments, making efficient simulations of molecular interactions with substrates highly challenging.

To address these limitations and provide an efficient tool for SPM manipulation and self-assembly of small organic molecules on ionic substrates, we have implemented a specialized grid-projected force field (GridFF) description into our classical force field simulation software FireCore.^34^ The GridFF method – which is the main focus of this publication – is admittedly inspired by what is used typically for rigid protein–ligand docking.^35–37^ Unlike traditional force fields, which evaluate all pairwise interactions on-the-fly, GridFF precalculates the molecule–substrate interaction potential on a grid before the actual simulation is performed. This precalculated grid, representing the interaction potential, is then simply interpolated during the simulation. Such an approach enables precise and computationally feasible predictions of molecular self-assembly and manipulation on inorganic surfaces, bridging the gap between theoretical modeling and experimental nanostructure design.

## 2 Methodology

As noted at the end of the previous section, grid-based potentials for the description of noncovalent interactions are routinely used in computational biochemistry codes for protein–ligand docking.^38–40^ Such approaches are well-established: the total interaction energy between a molecule and a substrate is typically decomposed into atomic contributions, which can be projected onto a grid and efficiently interpolated using 3D trilinear^41^ or tricubic splines.^42^ In the following, we organize our discussion into three aspects of GridFF: interpolation of the potential, projection of molecular interactions onto the grid, and treatment of electrostatics.

### 2.1 Interpolation

In ligand docking applications, the trilinear approximation prevails due to its minimal computational cost, despite the fact that it provides limited accuracy (even though, especially for coarser grids, interpolation errors for energy can be mitigated by using power transformations^43,44^ ). Grid potentials are then typically used to evaluate energy-based scoring functions only, rather than for molecular dynamics (MD) simulations or force-based optimizations.^45^ Moreover, these potentials are often artificially softened to effectively mimic protein flexibility and thermal fluctuations at room temperature.^43^ In some approaches, grid force fields are used to map free energy profiles via Monte Carlo sampling at finite temperature,^46–48^ where inaccuracies in energy and forces are smeared out by thermal fluctuations. In contrast, our GridFF applications target force-based dynamical simulations at low temperature, where trajectories closely follow the potential energy surface (PES), and a delicate balance between the PES gradient and the driving force (from the AFM tip) determines bifurcations near saddle points.

Another fundamental problem of trilinear interpolation is the inconsistency between interpolated forces and energy. In such implementations, the three force components and energy are stored and interpolated independently, which requires four times more memory, and produces forces that are not the exact gradients of the energy field.^49^ As a consequence, this may hinder force convergence in MD below a certain threshold, and/or prevent energy conservation. To resolve this, we implemented tricubic interpolation of the energy field, with forces obtained analytically as gradients of the piecewise cubic polynomials. In one dimension, this yields piecewise quadratic, continuous, and smooth force profiles. After testing several approaches, including Hermite cubic polynomials, we selected cubic B-splines, which provide the best trade-off between interpolation accuracy and computational performance.

High-performance implementations on modern CPUs and GPUs must optimize memory access and cache locality. The 3D cubic B-spline interpolation is implemented as a Cartesian (tensor) product of 1D interpolations. Each evaluation accesses a 4 × 4 × 4 block of the nearest grid points (64 values), and for each potential component (i.e., Coulomb, Pauli, and London), this requires reading 192 floating-point numbers. The grid is stored so that the fastest-changing axis (e.g., *z*) is contiguous, with blocks of four *z*-values collocated in memory. While a direct sum over 64 basis functions is possible, more efficient implementations – such as in FireCore^34^ – decompose the operation into a sequence of 1D interpolations: 16 along *z*, 4 along *y*, and 1 along *x*. Starting with the fastest axis (*z* in our case) maximizes cache locality and enables single-instruction-multiple-data vectorization.

Moreover, arithmetic operations are much faster than global memory reads on contemporary hardware, preloading nearby addresses in cache accelerates interpolation when the required data are stored in contiguous blocks. According to our benchmarks, the tricubic B-spline interpolation is roughly twice as slow as trilinear interpolation (using grids for energy and force components), while providing several orders of magnitude higher accuracy. Moreover, on CPU, the evaluation of molecule–substrate interactions using GridFF is still more than 10 times faster than the evaluation of pairwise nonbonded interactions within the molecule, making the extra cost negligible. On GPU, the GridFF interpolation consumes a significantly higher share of performance budget (see Section 6) as the GPU performance is limited by global memory access rather than arithmetic operations.

One complication of using B-splines is that the expansion coefficients stored at each grid point are not known *a priori* and must be fitted to reproduce reference values at grid nodes. Although such a fitting is a linear problem involving a very sparse matrix, the large grid size (∼10^6^ points per cubic nanometer at 0.1 Å spacing) favors iterative solvers over direct matrix inversion. Currently, we use a simple gradient descent algorithm to minimize the root-mean-square error across all grid points. While convergence may take thousands of iterations, we do not consider this as a bottleneck because grids are precomputed once per substrate and reused in many simulations. Consequently, we have not prioritized further optimizations of the coefficient fitting procedure.

### 2.2 Factorization

In its simplest form, a grid-based potential approximates the molecule–substrate interaction by projecting typical noncovalent pairwise potentials – such as Coulomb, Morse, or Lennard-Jones – onto the grid. This generally requires evaluating energy (and forces) contributions for each atomic type of the molecule interacting with all substrate atoms. While feasible in principle, this can be very memory demanding, as tens or hundreds of grids may be needed for all possible combinations of atom types in the molecule and in the substrate, each accounting for hundreds of megabytes of memory.

Cache efficiency further limits this approach, finally making this formulation practically useless. For this reason, grid-based potentials often^36,37,43^ (but not always^41,42,50^ ) rely on factorization schemes where parameters of the ligand atoms (the molecular adsorbate in our case) can be factored out of the summation over substrate atoms. While trivial for electrostatics (where the prefactor is simply the atomic charge), factorization of Pauli repulsion and London dispersion is more subtle. While in the literature are reported formulas for factorizing the Lennard-Jones potential with the geometric-mean mixing rules,^36,37,43^ such an approach is not viable if arithmetic-mean rules have to be used (which, for example, is the case for the AMBER^25^ and CHARMM^26^ force fields). Here, we show how to extend factorization to the Morse potential with Lorentz–Berthelot mixing rules (as used in the following). The nonbonded energy *E*_*i*_ of an atom *i* of the molecule at position *r*_*i*_ is

1 $$E_{i}\left(r_{i}\right)=\sum_{j}\sqrt{\varepsilon_{i}\varepsilon_{j}}\left[e^{-2\alpha \left(\left|r_{i}-r_{j}\right|-R_{i}-R_{j}\right)}-2e^{-\alpha \left(\left|r_{i}-r_{j}\right|-R_{i}-R_{j}\right)}\right]$$

where *ε*_*i*_(*ε*_*j*_) and *R*_*i*_(*R*_*j*_) are the depth of the energy well and the equilibrium distance for atom *i*(*j*), respectively, and the sum runs over all substrate atoms *j* (at position *r*_*j*_). The factorization can then be achieved simply by

$$E_{i}\left(r_{i}\right)={\sqrt{\varepsilon }}_{i}e^{2\alpha R_{i}}\underset{j}{\sum }{\sqrt{\varepsilon }}_{j}e^{-2\alpha \left(\left|r_{i}-r_{j}\right|-R_{j}\right)}-2{\sqrt{\varepsilon }}_{i}e^{\alpha R_{i}}\underset{j}{\sum }{\sqrt{\varepsilon }}_{j}e^{-\alpha \left(\left|r_{i}-r_{j}\right|-R_{j}\right)}$$ 2

since all terms within the sums over substrate atoms are independent of atom *i*. This allows any molecule to be simulated without further precomputation, and GridFF imposes no restriction on the number of substrate atom types.

### 2.3 Electrostatics

The treatment of long-range Coulomb interactions in classical force field simulations requires some attention. A naive evaluation of electrostatic energy via direct pairwise summation does not converge for periodic systems, and even in neutral systems, electrostatic forces converge only very slowly with distance, consuming a relevant amount of computational resources. Fortunately, periodic boundary conditions (PBC) allow the use of efficient reciprocal-space summation techniques based on the Ewald summation.^51^ For instance in the Particle-Mesh Ewald (PME) method,^52^ atomic charges are projected onto a grid as a charge density ρ­(*r⃗*), and the electrostatic potential is computed in reciprocal space via *V*(*k⃗*) = ρ­(*k⃗*) /|*k⃗*|^2^, using fast Fourier transforms (FFT) to switch between real and reciprocal space representations. This computational cost scales as *O*(*N* log (*N*)) with the number of grid points *N*.

Efficient PME implementations split the charge density into a smooth (low-frequency) component solved in reciprocal space and a high-frequency residual handled in real space, allowing the use of relatively coarse grids. While sophisticated smoothing and splitting schemes exist,^53^ details are beyond the scope of this article. Nevertheless, even with these optimizations (imposing PBCs on the system, using highly optimized PME solvers and FFT libraries), electrostatics still accounts for a significant portion of the computational cost in classical MD simulations. Moreover, due to its efficiency, PME is often used even for systems that are not naturally periodic (e.g., proteins in water, or a single molecule on a surface), at the cost of introducing artificial interactions between periodic images.

Here, GridFF offers major advantages in both speed and accuracy. We split the system into two subsystems (i) substrate and (ii) molecule and treat them separately. The Ewald method is used only for the substrate where PBCs are physically justified. Thanks to the rigidity of the substrate, the potential of the substrate is precomputed, entirely eliminating the computational cost of solving it (via PME, or any other method) from the MD run. The molecule interacts with the substrate subsystem exclusively via interpolation of this precalculated surface potential. The noncovalent interactions within the molecular subsystem are computed by direct sum (in real space) without imposing unphysical PBCs, thus eliminating spurious interactions between periodic images of the molecule, which are otherwise introduced by traditional methods (such as by applying the PME method to the whole system).

Moreover, in fully periodic systems (e.g., a pristine surface), all interactions can be folded into the unit cell of the substrate, even if the molecular adsorbate is much larger, and therefore storing all interaction data in a minimal grid. During the MD run, the atomic coordinates of the adsorbate are then mapped into the surface unit cell and used for computing energies and forces.

In addition, for periodic surfaces without defects, the surface potential becomes negligible just a few Å above the substrate, as both Morse and electrostatic components decay exponentially with the height. For example, a pristine NaCl substrate can be stored in as little as 32 Å^3^ or 256 kB per component. For nonperiodic systems (e.g., point defects, step edges, AFM tips), memory requirements are higher, e.g., 100 nm^3^ and 800 MB per component in our largest 20 × 20 supercell. Overall, aside from the assumption of surface rigidity, memory usage is the main limitation of GridFF.

In this work, we test the GridFF approach using a simplified electrostatic solver that omits the real-space residual entirely and relies solely on solving the Poisson equation ∇^2^ *V* = ρ in reciprocal space. Thanks to the fine grid spacing used (0.1 Å), this achieves ∼0.01 meV accuracy, sufficient for most purposes. Nevertheless, in future work, we plan to implement the full PME algorithm including the real-space correction to further improve accuracy. In this respect, our FireCore^34^ implementation more closely resembles the electrostatic solvers used in density functional theory (DFT) codes (e.g., SIESTA^54^ or VASP^55^), rather than those based on classical force fields like LAMMPS. Besides simplicity, our motivation for this approach is the possibility to generate GridFF directly from *ab initio* charge densities without requiring atomic charge assignment or projection. In fact, GridFF does not necessarily need to be constructed from pairwise potentials (e.g., as in the D3 van der Waals corrections,^56^ where three-body terms are considered), but can also be computed directly from electron densities obtained from *ab initio* wave functions, as it is routinely done in the so-called full density based model (FDBM) in AFM simulations.^57,58^

## 3 Accuracy and Performance Tests on CPU

In order to benchmark the accuracy of the proposed approach, we performed several systematic tests calculations using GridFF as implemented in FireCore^34^ and all-atom simulations using the LAMMPS package.^32^ The studied system is the desorption and manipulation of a 3,4,9,10-perylenetetracarboxylic dianhydride (PTCDA) molecule on top of a NaCl slab. This system was chosen as it has been extensively studied experimentally by means of SPM techniques.^59–61^ Also robotic manipulation of the PTCDA molecule (although on metallic substrate) originally motivated our work.^18^

First, we performed rigid (i.e., with fixed relative positions of the atoms in the molecule) vertical and lateral scans of PTCDA on a 8 × 8 × 3 NaCl(001) surface slab to compare individual potential components between GridFF and LAMMPS. Next, we conducted relaxed scans with the same molecular and substrate configurations, allowing the system to undergo molecular relaxation, therefore more closely mimicking a manipulation experiment with AFM. After validating the relaxed potential behavior, we extended our tests to include defective substrates. We introduced a neutral, nearly isolated defect by removing 2 atoms from the surface of a 20 × 20 × 3 NaCl supercell, equivalent to a defect density of approximately 0.08%. Using the same PTCDA molecule, we performed lateral scans across this defected surface to test the capability of the method to handle surface irregularities. Finally, we compared the performance of GridFF and all-atom calculations for increasing substrate sizes ranging from 8 × 8 × 3 to 20 × 20 × 3 supercells.

The molecular structure of PTCDA was retrieved from a previous work of one of us on high resolution AFM imaging,^58^ and supplemented with atomic charges determined using the restrained electrostatic potential (RESP) fitting scheme.^62^ The NaCl substrate was modeled by three *fcc* atomic layers with lattice spacing of 4.0 Å, and charges for the Na/Cl ions set to ±0.9e according to the Bader analysis.^63^ The intramolecular interactions are modeled using the universal force field (UFF)^64^ both in FireCore and LAMMPS. UFF values were also used to obtain the Morse parameters modeling the molecule–substrate interaction (the value of the α parameter was set to 1.5 Å^–1^ for all pairs, and a cutoff of 17 Å was applied).

Within the GridFF framework, we generated potential energy grids for the NaCl(001) substrate using a fine grid spacing of 0.1 Å and setting the maximum iterations to 3000 for the generation of the B-spline parameters, achieving fitting errors on the order of 10^–5^ for both Morse and Coulomb potentials. For the treatment of the slowly decaying electrostatic interactions, in FireCore we used a reciprocal-space Poisson solver as reported above, while in LAMMPS they were evaluated with the closely related particle–particle particle-mesh (PPPM) method^65^ with a real space cutoff of 15 Å. The relative accuracy of reciprocal part of PPPM kernel was set to 10^–8^ in rigid scans to obtain high-accuracy reference energy profiles. Such a stringent tolerance was necessary since a more standard setting (10^–6^) produced numerical artifacts in LAMMPS calculations larger than the error coming from the reciprocal-space-only Poisson solver in GridFF. Nevertheless, a more relaxed threshold of 10^–6^ was chosen for relaxed scans with LAMMPS, as these simulations were also used for benchmarking the performance in typical use-cases. Therefore, we should note that in case of relaxed scans FireCore simulations are actually more accurate then LAMMPS results, while being significantly faster. To ensure a fair comparison, both FireCore and LAMMPS used the same FIRE algorithm^66^ for the structural optimization with a convergence threshold for forces of 10^–3^ eV/Å.

### 3.1 Rigid Scans of PTCDA on NaCl

The profiles in Figure 1 show the contributions to the energy variations when a PTCDA molecule is rigidly moved away from the NaCl surface. The decomposition of the total interaction potential into individual components ensures that the underlying interaction physics is accurately reproduced by the GridFF approach. The Morse potential component (red) describes the short-range interactions (encompassing both Pauli repulsion and van der Waals attraction), while the Coulomb potential component (blue) captures the long-range electrostatics between the PTCDA molecular charge distribution and the ionic NaCl substrate. The total potential energy curve (green) represents the superposition of all interaction components, yielding an equilibrium adsorption configuration at *Z* = 3.1 Å with a total binding energy of −0.89 eV. All energy profiles overlap perfectly, and therefore, to quantitatively estimate the accuracy of GridFF approach, we also report the energy differences between the two methods on the right *y*-axis. In all cases, the agreement is remarkable (considering the fitting error in the interpolation procedure, and the PPPM accuracy set in the LAMMPS calculations), with the Morse potential component exhibiting differences on the order of 10^–6^ eV, while the Coulomb component shows discrepancies of approximately 10^–5^ eV. Overall, the differences are dominated by the electrostatics contribution, due to the plane-wave cutoff of the reciprocal Poisson solver and the aforementioned omission of real-space residual from PME employed in the GridFF calculations. In both cases, the differences tend to increase at small separation distances (where the slope of the profiles is maximal in absolute values).

**Figure 1:**
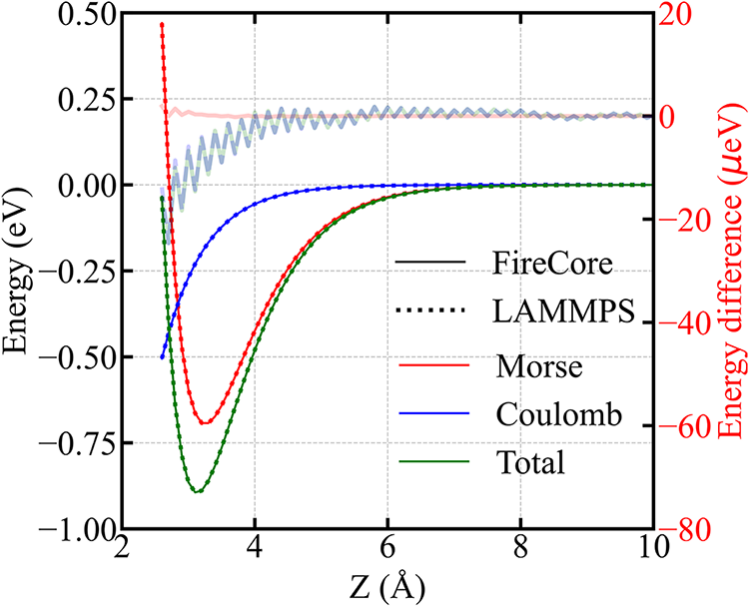
Energy profiles for a PTCDA molecule interacting with a NaCl surface as a function of the separation distance along the *z*-direction. The total potential is decomposed into Morse (red) and Coulomb (blue) components, with the total energy (green) shown for the sake of completeness. Calculations using the GridFF approach as implemented in the FireCore code are reported with thin solid lines, while all-atom simulations from LAMMPS are in thick dashed lines. The energy differences between the two methods are plotted with semitransparent lines and with the same color scheme.

Next, we performed lateral two-dimensional rigid scans on the *xy*-plane by rigidly displacing the PTCDA molecule with a step length of 0.1 Å and at a constant height of 3.3 Å from the surface. Figure 2 shows the total interaction energy as a function of the in-plane position of the molecule across one unit cell of the NaCl(001) substrate. Such a 2D scan provides information about the preferred adsorption sites and barriers for lateral diffusion or manipulation of the molecule on the surface. The left and center panels show the PES calculated by FireCore and the reference LAMMPS force field, respectively. Both methods produce a qualitatively identical energy landscape, with an energy barrier of 0.46 eV located at the center of the unit cell. The right panel displays the absolute energy difference between the FireCore and LAMMPS calculations. As in the previous case, the absolute error is on the order of 10^–5^ eV. In summary, these results demonstrate that FireCore accurately reproduces the whole interaction energy landscape.

**Figure 2:**
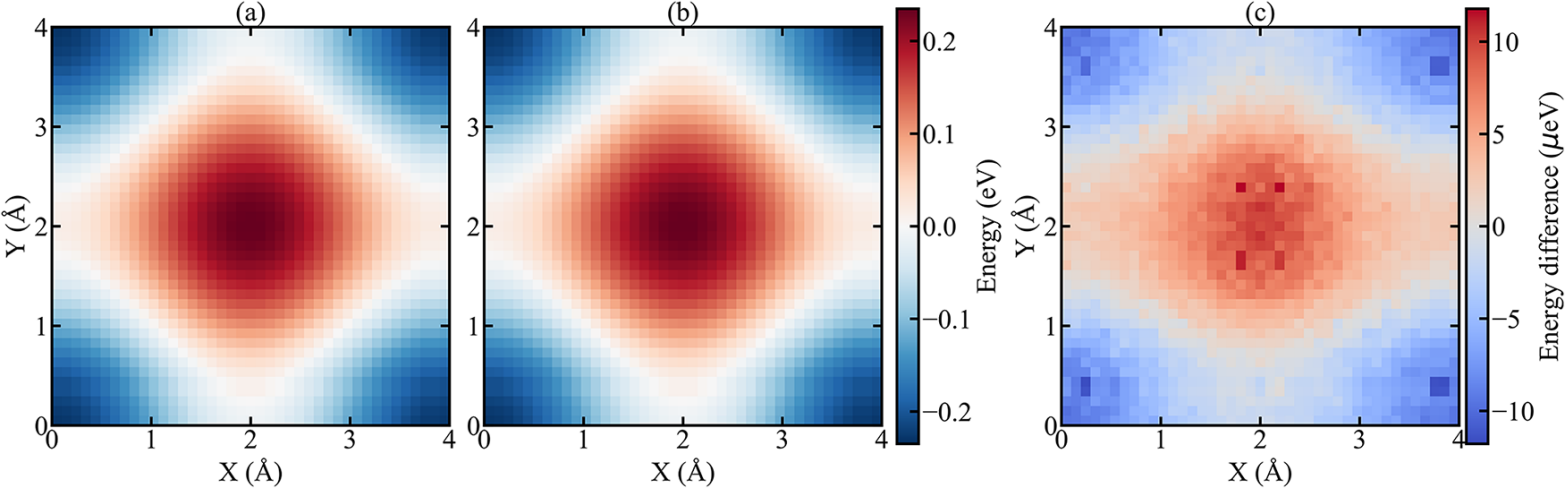
Rigid PES of a PTCDA molecule interacting with a NaCl surface at a separation height of 3.3 Å, obtained with the GridFF approach as implemented in the FireCore (a), and all-atom calculations from LAMMPS (b). Panel (c) reports the energy difference between FireCore and LAMMPS.

### 3.2 Relaxed Detachment of PTCDA from NaCl

In order to simulate more realistically the manipulation of PTCDA with an SPM tip on a surface at low temperature, we performed calculations where we restrained the position of one atom (a carboxylic oxygen at the corner) of the molecule and let the position of the other atoms relax. In the first set of simulations, at each step we displaced the *z*-component of the position of the selected atom by 0.1 Å, starting from 1.3 to 20 Å above the NaCl(001) surface. Such a computational setup effectively mimics the experimental protocols where a functionalized tip is used to grasp and manipulate individual molecules on surfaces by forming a mechanical contact with a specific atomic site.^17,18^ The resulting relaxed potential energy curve reported in Figure 3 exhibits significantly different characteristics compared to the rigid scan. The global minimum occurs at approximately *Z* = 2.8 Å with a binding energy of −0.94 eV, representing the equilibrium adsorption configuration. The larger binding energy (compared to the rigid scan) demonstrates the importance of molecular flexibility in achieving optimal molecule–surface interactions through conformational adaptation. The unbinding energy profile is also qualitatively different, presenting multiple local minima and discontinuities, particularly in the intermediate separation range (6–15 Å). These features arise from the interplay between attractive molecule–surface interactions and internal molecular strain. The molecular relaxation allows for rotation, tilting, and conformational changes (that can create metastable adsorption states not accessible in rigid scan calculations), and eventually leading to the sudden detachment of the molecule from the substrate. As already pointed out, in the proximity of the sudden changes of molecular orientation/conformation, the relaxation dynamics is more sensitive to small force differences, leading to bifurcation in the taken path. Nevertheless, also in this case the quantitative agreement between FireCore and LAMMPS is good, with energy differences remaining below 0.9 meV across the entire scan range. These results validate the ability of the GridFF approach to accurately capture the energetics and the structural response of a molecule under mechanical manipulation, a critical requirement for predicting AFM-based molecular device fabrication and single-molecule manipulation protocols.

**Figure 3:**
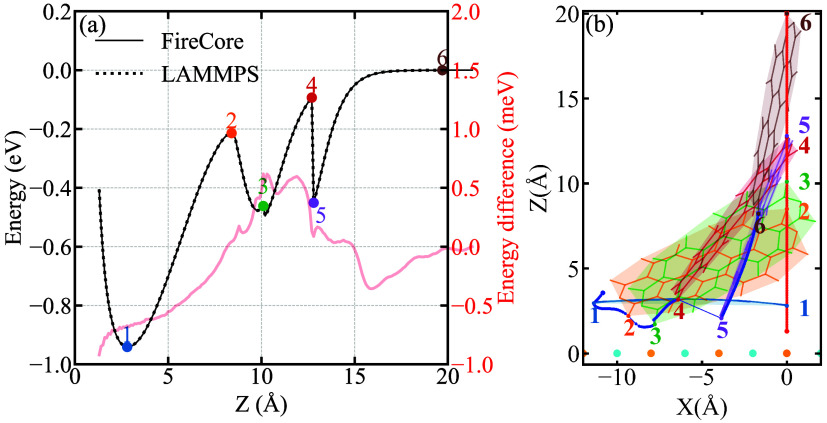
(a) Energy profiles for both GridFF (FireCore) and all-atom (LAMMPS) calculation, and (b) selected configurations of a PTCDA molecule lifted up from a NaCl surface. In panel (b), the red and blue dots represent the atom that is displaced vertically during the scan, and the carbonyl oxygen at the opposite position of the PTCDA molecule, respectively. The six molecular configurations shown in (b) correspond to the marked dots in (a). Na and Cl ions are depicted in orange and cyan, respectively (only the topmost layer is shown).

### 3.3 Dragging the PTCDA Molecule over a Defect

The goal of this set of simulations is to mimic the dragging of a PTCDA molecule by an SPM tip over a NaCl surface along the diagonal of the substrate cell. As done before, this was achieved by fixing the position of one atom of the PTCDA molecule (marked by the red dot in Figure 4), and systematically displacing it along the scan direction while allowing the entire molecular system to relax at each step. We have considered a 20 × 20 × 3 NaCl substrate with 2400 atoms for the pristine surface, and a system with a neutral defect (i.e., by removing one Na and one Cl atoms next to each other from the topmost atomic layer) placed at the center of the cell.

**Figure 4:**
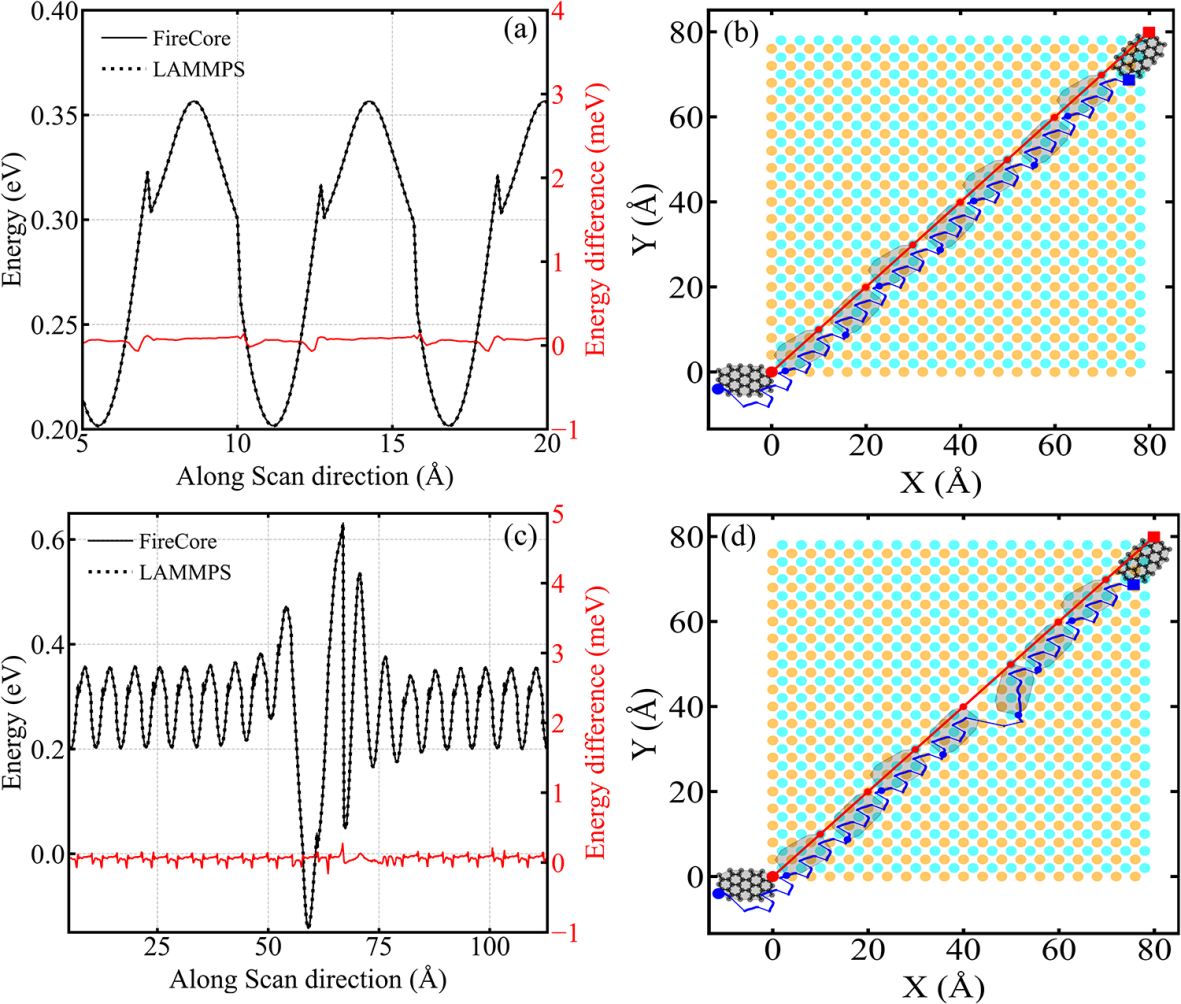
Comparison between GridFF (FireCore) and all-atom (LAMMPS) calculations for a PTCDA molecule dragged over a NaCl surface with and without the presence of a defect. (a) Energy profile and (b) the corresponding set of relaxed configurations on a pristine 20 × 20 × 3 NaCl substrate. (c) Energy profile and (d) the corresponding set of relaxed configurations on the same substrate in the presence of an isolated neutral defect at the center of the cell. In the energy plots (a, c), the potentials calculated by FireCore (dashed orange) and LAMMPS (solid blue) are shown on the left axis, with their absolute difference (red) on the right axis. In the trajectory plots (b, d), the path of the restrained atom (red) and of the atom in the opposite corner of the molecule (blue) are shown with dots, illustrating the path taken by the molecule over the Na (orange) and Cl (cyan) ions of the top substrate layer.

The top panels of Figure 4 illustrate the sliding behavior on a defect-free NaCl surface. The energy profile in Figure 4a shows a quantitative agreement between FireCore and LAMMPS, with the difference remaining consistently below 0.9 meV. The energy minima correspond to the PTCDA molecule settling into energetically favorable adsorption sites that align with the underlying Na and Cl ion lattice. The energy maxima represent the potential barriers that the molecule must overcome to move between these stable sites. The nonsmooth nature of the energy profile arises from the interplay between the static interaction field generated by the substrate and the orientational and conformational degrees of freedom of the PTCDA molecule. As it is pulled across the surface, the molecule continuously adjusts its orientation and internal geometry to minimize the total energy, leading to the characteristic “stick–slip” motion (reminiscent of different systems studied by AFM experiments and MD simulations^67,68^ ) visually depicted in the trajectory plot (Figure 4b). The path of the unconstrained opposite corner atom (blue dot) clearly deviates from the straight-line path of the fixed atom, hopping between adjacent lattice sites.

The bottom panels of Figure 4 shows the capability of FireCore to handle localized and chemically complex features, such as a neutral point defect in the substrate. It can be observed from the graph that, far from the defect, the energy profile in Figure 4c retains the periodic corrugation of the pristine surface. However, as the molecule approaches the defect location (at the midpoint of the scan length), the energy profile changes drastically. A sharp, deep potential well emerges, indicating a strong pinning of the molecule to the defect site. This interaction is significantly stronger than the regular surface corrugation, with an energy stabilization of over 0.77 eV. Figure 4d provides an intuitive real-space visualization of this event: the path taken by the molecule shows a lateral deviation as it is influenced by the presence of the defect. The PTCDA molecule reorients itself to maximize its favorable interaction with the defect before being pulled away, which requires overcoming a substantial energy barrier. Also in this case, the energy profile obtained with GridFF perfectly matches the one from reference calculations, with maximum deviations in the order of 0.9 meV. Overall, this demonstrates the capability of the proposed approach to accurately model the molecule–surface interaction even in the presence of more complicated features such as a point defect, making it a powerful tool for predictive materials simulation.

### 3.4 Performance Comparison

The lateral relaxed scan on the pristine surface described in the previous section was also used to benchmark the performance of the CPU implementation of GridFF in FireCore with respect to LAMMPS. The dynamical relaxation with the FIRE algorithm^66^ is a good choice for such comparison, as the overheads (e.g., setup and initialization of the system) are amortized over the thousands of steps required for the structure relaxation. To ensure a reliable comparison between the two approaches, and avoid any dependence on the details of the relaxation algorithm, the number of relaxation steps was set to the same value (5000), and with a tiny threshold on the force convergence criterion to ensure that the maximum number of relaxation steps is always reached. In this way we can directly compare the calculation walltimes needed to complete the dragging path, as in both cases the same number of relaxation steps (and therefore of force and energy evaluations) are performed. It must be stressed that, in order to have a fair comparison, the PPPM accuracy tolerance in LAMMPS was increased to 10^–6^ (with respect to 10^–8^ used for rigid-scan calculations). Moreover, again for the sake of fairness, we performed calculations also directly excluding the computation of pairwise interactions within the substrate, by removing the appropriate atoms from the neighbor lists.

Figure 5 shows the comparison of the computational performance between LAMMPS and Firecore, together with the scaling with respect to the system size (ranging from 8 × 8 × 3 to 20 × 20 × 3 NaCl unit cells, corresponding to 384 to 2400 substrate atoms). All calculations were performed on the same computer equipped with an AMD EPYC 7513 CPU (2.6 GHz) using a single core. FireCore achieves good speedup factors ranging from 113× for the smallest system to 751× for the largest, reducing execution times from hours to minutes, as also shown in Table 1. Notably, the largest system, that requires over 45 h in LAMMPS, completes in less than 4 min with FireCore. Moreover, as one can notice from the different slopes in Figure 5, GridFF shows also a superior scaling behavior with respect to the system size, if compared to all-atom calculations. In fact, by moving from the smallest to the largest systems, the total number of atoms increases by about 6 times. Correspondingly, the execution time (normalized by the total number of path points) increases by almost 8 times in LAMMPS, while in FireCore the increment is only about 20%. Such dependence of speedup factors on the system size indicate that the algorithmic advantages of GridFF may become even more pronounced for larger systems (such as pulling of graphene ribbons^3^ or DNA), enabling practical high-throughput screening and statistical sampling for several applications.

**Figure 5:**
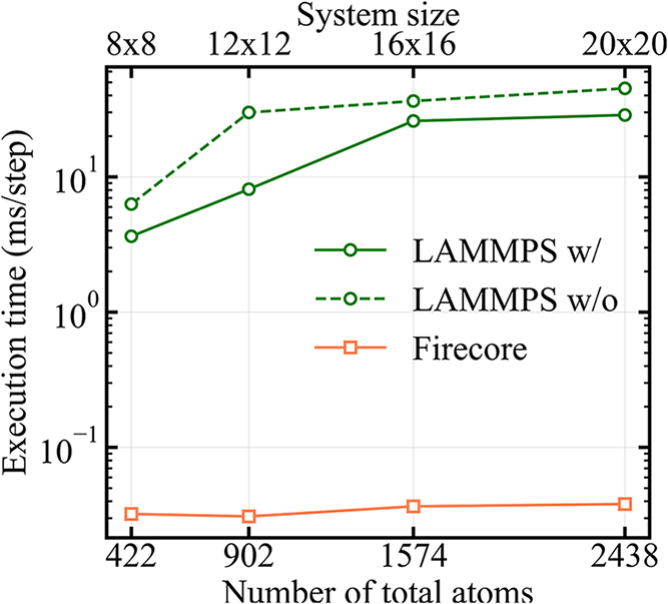
Execution walltimes for FireCore and LAMMPS as a function of the system size. The green solid and dashed lines represent LAMMPS calculations with and without excluding the computation of pairwise interactions within the substrate atoms, respectively.

**Table 1:** Execution Times and Computational Speedups for FireCore and LAMMPS for Different System Sizes

			LAMMPS			
			with exclusion		without exclusion	
system size	total steps	FireCore time (s)	time (s)	speedup	time (s)	speedup
8 × 8 × 3	2,265,000	73	8,240	113	14,256	195
12 × 12 × 3	3,395,000	105	27,549	262	101,655	968
16 × 16 × 3	4,525,000	166	117,259	706	164,526	991
20 × 20 × 3	5,660,000	216	162,257	751	255,390	1,182

## 4 Configuration Sampling on GPU

While the simulations of dragging of the PTCDA molecule using CPU allows a side-by-side comparison of the accuracy and performance of GridFF/FireCore with LAMMPS, the main strength of our approach lies in its ability to accelerate the exploration of large configuration spaces of flexible molecules on surfaces. This includes tasks such as finding the most stable binding configuration (i.e., the global energy minimum in the configuration space, which is a hard problem), or computing the binding potential of mean force, which requires sampling all energetically relevant configurations.

To illustrate the performance of FireCore in such applications, we conducted a case study of the adsorption of a xylitol molecule on a sodium chloride surface with a single chlorine vacancy. The presence of five hydroxyl groups in the xylitol molecule creates numerous possibilities for hydrogen bonding with the ionic surface. Combined with molecular flexibility due to free rotations around sigma bonds, this results in a complex energy landscape characterized by many local minima.

For the simulation we used our experimental sp3FF force field, which is currently under development and it will be published separately later. At the moment, sp3FF is the only force field fully implemented on GPU in FireCore (porting UFF is in progress). The sp3FF force field represents an attempt to simplify and optimize UFF (and similar valence-based force fields) for parallel architectures. The main idea is to replace explicit 4-body interactions (dihedrals and inversions), with 3-body π-π and π-σ terms between the orientation vectors of atoms (which represent *p* orbitals, and are added as additional degrees of freedom) and bonds. This modification reduces the amount of synchronization between threads when writing force components to the atoms involved in the interaction. Nevertheless, in the case of the xylitol molecule, the differences between UFF and sp3FF are rather irrelevant, due to the absence of any inversion or π-orbital, and negligible barriers for rotation around sigma bonds. Therefore, we expect only minor differences between UFF and sp3FF also in terms of performance, as the results reported in Figure 6c show that the computational cost is dominated by nonbonded interactions with substrate. The aim of this case study is not to provide realistic adsorption geometries for the particular system or potential, but rather showcase the general application of GridFF for efficient exploration of the vast configuration space of a flexible molecules on a surface. In particular, it illustrates the ability to run many (thousands) replicas of the system in parallel using a single GPU. As such, the results are general, independently of the exact formulation of the bonded potential employed.

**Figure 6:**
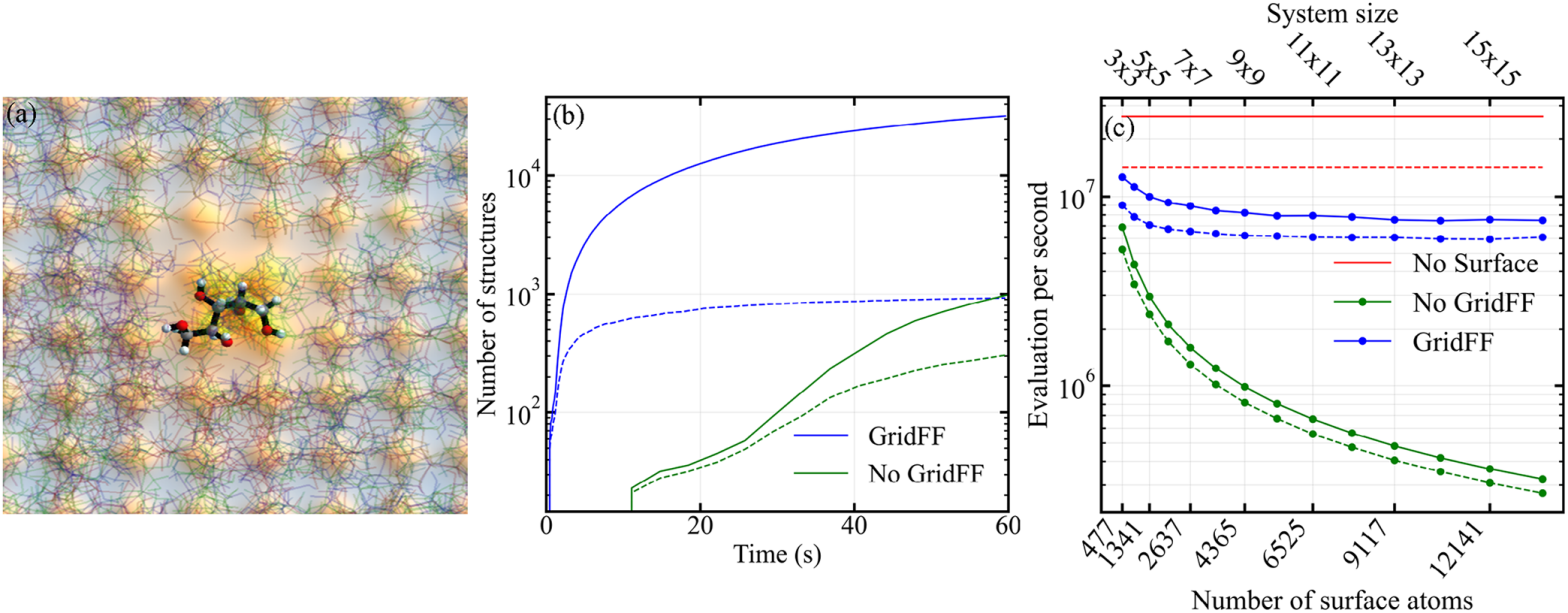
Analysis of xylitol adsorption on NaCl using GridFF. (a) Visualization of a 2000-replica simulation of representative xylitol molecule configurations adsorbed on the NaCl(001) surface with a Cl-hole. (b) Number of total (solid) and unique (dashed) structures found in a short run using FireCore. Blue and green lines represent the outcomes of simulations done on 16 × 16 × 3 surface with a Cl-hole using the GridFF and standard all-atom approaches, respectively. (c) Number of MD steps per second using GridFF (blue) and all-atom (green) calculations, and for an isolated xylitol molecule (red) for reference, as a function of the system size. Solid and dashed lines corresponds to calculations with different settings: 5000 replicas and configurations downloaded to the CPU every 100 steps for the former, and 1000 replicas and configurations downloaded every 20 steps for the latter.

To comprehensively explore this vast conformational space, we employed a minima hopping technique adapted for surface adsorption. This method systematically samples different configurations by perturbing the molecular structure i.e., by performing 1000 steps of Langevin MD at a relatively high temperature of 300 K, followed by dynamical relaxation to the nearest local minimum. Each energy minimization was carried out until forces converged below 0.1 meV/Å, ensuring an accurate representation of the stable configuration.

Efficient implementation of MD for small molecules like this on GPU faces several challenges. Modern GPUs are equipped with thousands of cores, which is significantly more than the number of atoms in such systems (typically 50–100 atoms; rigid substrate atoms represented by GridFF are excluded). Although parallelization over individual pairwise interactions (rather than atoms) is possible, synchronized output of forces (i.e., reduction) from different threads to global memory would require thread synchronization, reducing performance. We address this challenge by simulating multiple replicas of the same system (i.e., conceptually molecule and substrate) in parallel.

Technically, this is implemented using 2D OpenCL kernels, where the first dimension assigns GPU threads to individual atoms in each replica, while the second one parallelizes over replicas. The first dimension is further split into workgroups of 32 threads (optimal for NVIDIA GPUs) which share fast local memory. If the number of atoms in the system is not divisible by 32, the last workgroup is padded with idle threads. We found that this is generally still more efficient than using smaller workgroups, although there is room for detailed benchmarking for different systems. This is done for all kernels (i.e., all replicas are processed by a single kernel call), which reduces the number of kernel calls and associated overhead. An iteration of the MD loop consists therefore of 3–4 kernel calls. When using GridFF, we combine the evaluation of nonbonded molecule–molecule and molecule-grid interactions into a single kernel getNonBond_GridFF_Bspline to reduce the number of kernel calls. If the substrate is represented by atoms rather than a grid, the kernels getNonBond and getSurfMorse are called separately. All such kernels use the fast local memory within the workgroup to preload atomic coordinates and force field parameters, and evaluate pairwise interactions in blocks of 32 × 32 atoms to minimize global memory overhead. The kernel getMMFFf4 evaluates bonded forces (bonds, angles, and eventually π-π or π-σ interactions) including recoil forces (due to the Newton’s third law) on bonded neighbors, which are stored in separate memory slots (to avoid asynchronous writes) for being assembled later. Since both 2- and 3-body interactions are local for a given atom, the parameters are stored directly in the registers or in the private memory of a given thread and it does not need to be shared. The kernel updateAtomsMMFFf4 assembles the recoil forces, applies the Langevin thermostat or velocity damping, and then updates atomic coordinates (and orientation vectors) using leapfrog integration. During the MD loop, each replica keeps its own instance of thermostat and optimization parameters, therefore, at a given time, each one can be in a different phase of the minima hopping process (i.e., some replica may be thermally excited, while others are being relaxed to the local minimum). Notice that while the substrate is rigid and identical for all replicas, substrate atoms are excluded from the dynamics, and only a single instance of GridFF or surface atoms is stored in the global memory and shared by all replicas in getNonBond_GridFF_Bspline and getSurfMorse kernels.

All calculations were performed on a desktop NVIDIA RTX 4060 Ti GPU. Testing showed that the optimal performance is achieved with ∼5000 replicas, in the case of the xylitol molecule. Moreover, the time required to evaluate forces (i.e., summing over all bonding and noncovalent interactions) for such small systems is often shorter than the time required to transfer atomic coordinates (and forces) to and from the GPU. Therefore, the entire MD loop – including force evaluation and integration of the equations of motion – must be executed entirely on the GPU, eliminating the need for costly synchronization with the CPU.

While Langevin dynamics is performed fully on the GPU and downloaded only every few hundred steps for visualization, the dynamical relaxation requires global properties which involve reductions over all atoms – i.e., thread synchronization. In particular, for dynamical relaxations, one needs to calculate the norm of the whole force vector *F⃗* in order to check the force convergence criterion, and to set to zero all velocities (*v⃗* = 0) if the system inertially moves up the hill (⟨*F⃗* | *v⃗* ⟩< 0). To handle this, we download the system state after a certain number of steps and perform these operations on the CPU (although in principle, such reductions could also be performed on the GPU but at the cost of more complicated kernels). At any rate, not performing the check at every step does not seem to significantly hamper the overall performance of the algorithm. We attribute this to the smoothness of the trajectory near the minimum (which often is a long narrow “valley”) and to the fact that a relaxation typically takes several thousand steps anyway.

To identify unique structures, each optimized geometry is compared with all previously minimized structures using a root-mean-square deviation criterion (with a threshold of 0.1 Å), accounting for both conformational differences and variations in adsorption site and orientation relative to the surface. For this purpose, geometries are downloaded from the GPU and the comparison is performed on the CPU, typically only once per several thousand steps. Although the computational cost of comparison with hundreds or thousands of previously found local minima is substantial (using only CPU), it is amortized in the dominating cost of thermalization and relaxation, and therefore we did not attempt to implement this subtask on GPU yet.

The simulation can be run either as a batch calculation via the FireCore Python API – suitable for supercomputer production runs – or through a graphical user interface (GUI), useful for debugging and educational purposes on desktop computers. Figure 6a shows a screenshot from such an interactive simulation, highlighting a selected configuration near the vacancy, while the other replicas are shown as transparent skeletons in the background.

In Figure 6b,c we present performance metrics for this simulation using both the GridFF approach and a naive all-atom FireCore GPU MD loop implementation. In the all-atom simulation, molecule–substrate interactions are computed as a direct sum of pairwise interactions (i.e., without Ewald summation as used in LAMMPS). These are summed over the nearest periodic images of the surface supercell, while the interactions between surface atoms are omitted. Figure 6c reports the total number of substrate atoms interacting with the molecule (i.e., the number of atoms in the supercell plus its 9 periodic images used for the direct-sum evaluation). Notably, GridFF-based calculations are not only significantly faster but also more accurate, owing to the use of Ewald summation in constructing the GridFF.

Figure 6b presents a quantitative analysis of the sampling efficiency, showing the number of converged and unique structures found as a function of the execution time. The number of converged structures increases proportionally with the number of attempts, indicating the consistent performance of the energy minimization protocol. In contrast, the number of unique structures rises rapidly at early times and plateaus around 800 structures, despite continued sampling. This saturation suggests that our approach exhaustively explored the relevant conformational space of the xylitol-NaCl system under the chosen computational model.

Looking closely at Figure 6b, we observe that, in the all-atom simulation, the first batch of structures is found only after ∼10 s, reflecting the time required for the initial thermalization. This synchronization delay also explains why the green curve deviates from a steady-state sampling behavior around 20 s after the simulation start. A similar behavior is seen in the GridFF simulation, but it is compressed into the first 1–2 s due to its ∼20× higher performance.

Figure 6c provides a systematic analysis of how performance scales with system size. GridFF shows clear performance superiority for larger supercells, achieving a throughput of approximately 7.5 million MD steps per second for the largest systems, compared to only 0.3 million steps per second for the all-atom approach. Notably, GridFF performance remains stable regardless of the system size, while the all-atom approach degrades steadily with system size, making the performance advantage of GridFF even more pronounced for larger systems.

For small supercells (<200 atoms in the supercell, < 1800 atoms in 9 images), GridFF shows a slight increase in performance, likely due to an improved cache efficiency as neighboring voxel memory addresses are more contiguous. This effect could potentially be exploited further by using coarser grids.

## 5 FireCore from the User Perspective

FireCore is written in C++ and OpenCL languages, with python binding providing a convenient scripting interface. The generation of the surface potential can be done internally on CPU, or using a standalone pyOpenCL accelerated python script, which takes the structure of the substrate (provided by the user in the xyz or mol format), and the desired voxel size for the grid, and it generates and stores the grid potential. The syntax for generating the grid potentials presented in this paper is

python3 generate_grid.py

The main simulation engine of FireCore can perform energy optimization or MD calculations of molecules on surfaces. To start a run, is sufficient to provide the geometries of the molecule and the substrate. Supported formats are xyz and mol. In the case that a plain xyz file is provided for the molecule, FireCore automatically detect the bonding topology. FireCore accordingly assigns the atomic types and all parameters needed for bonded and nonbonded interactions. Two force fields are currently available in FireCore: sp3FF on both CPU and GPU, and UFF on CPU (porting UFF to GPU is in progress).

The user can also directly customize the parameter files for bonded and nonbonded interactions. Next, the user can decide whether to run grid-based or all-atom simulations. A folder (which also contains a README file with commands and explanations) with all files needed to run the examples reported in this paper is available in the FireCore repository.^34^ To reproduce the calculations presented in Section 3, one can run the following command:

python3 generate_scans.py

Along with the command line interface, FireCore also provides a GUI for running interactive simulations, real-time visualization of structural relaxations and MD runs, including the multiple-replica feature described in Section 4. The GUI allows the user to visualize atomic types, charges, and electrostatic potentials. It also enables the interactive manipulation of molecules by picking and dragging atoms with the mouse (resembling what is done in AFM manipulation of molecules). The GUI can be run using the command:

bash FireCore/tests/tMolGUIapp/run.sh

## 6 Conclusions and Outlook

In this work, we have demonstrated that grid-projected force fields – originally inspired by methods used for ligand docking in molecular biology – can be successfully applied to surface science, particularly for simulating molecular adsorption and manipulation on surfaces using SPM at low temperatures. As implemented in the new open-source simulation package FireCore, the method achieves a speedup of 2–3 orders of magnitude (compared to conventional all-atom simulations) for a PTCDA molecule on a NaCl surface using a single CPU, while maintaining a high accuracy of the results. We further showcase the CPU and GPU implementations of the method in FireCore which enables sampling of millions of molecular configurations per second, making it possible to exhaustively explore all local minima of small flexible molecules (e.g., xylitol) within just a few minutes on a standard desktop GPU. This is achieved by simulating thousands of system replicas in parallel within each kernel call, minimizing the overhead for small systems (made of tens or hundreds atoms) resulting from the elimination of surface atoms due to use of GridFF.

The main limitation of the method is the assumption of a rigid substrate. However, this is a common approximation even in traditional all-atom simulations, particularly due to the lack of accurate force fields for ionic crystal surfaces. This limitation can be partially mitigated by adjusting force field parameters to emulate the effective (softened) potential resulting from substrate atom deflections – a strategy commonly employed in ligand docking.^43^ In a future work, we aim to address this limitation more rigorously by incorporating additional force field components that allow for local polarization and substrate deflection, based on linear response theory.^69–71^ We are also developing a hydrogen-bond correction scheme within the GridFF framework, as well as density-derived potentials such as FDBM,^57^ which promise to deliver accuracy far beyond traditional pairwise interactions, like Coulomb, Morse, or Lennard-Jones, while retaining unparalleled computational performance. Although in this study we opted for a conservative approach to GridFF interpolation using cubic B-splines with very fine grid spacing, we recognize opportunities to further optimize the memory footprint and potentially improve the speed via a better cache locality. This could be achieved by experimenting with alternative interpolation strategies, including power-transformed interpolations,^44^ different forms of potential factorization, or nonuniform grid spacing schemes.^72^
